# The Enhancement of Fungal Disease Resistance in Major Staple Crops Using CRISPR-Cas Technology

**DOI:** 10.3390/genes16111263

**Published:** 2025-10-26

**Authors:** Zagipa Sapakhova, Rakhim Kanat, Dias Daurov, Ainash Daurova, Malika Shamekova, Kabyl Zhambakin

**Affiliations:** 1 Institute of Plant Biology and Biotechnology, Almaty 050040, Kazakhstan; 2 Tanir Research Laboratory, Almaty 050060, Kazakhstan

**Keywords:** CRISPR-Cas, fungal diseases, main agricultural crops, cereals, legumes, vegetables

## Abstract

Fungal pathogens represent a major constraint to global agricultural productivity, causing a wide range of plant diseases that severely affect staple crops such as cereals, legumes, and vegetables. These infections result in substantial yield losses, deterioration of grain and produce quality, and significant economic impacts across the entire agri-food sector. Among phytopathogens, fungi are considered the most destructive, causing a wide range of diseases such as powdery mildew, rusts, fusarium head blight, smut, leaf spot, rots, late blight, and other fungal pathogens. Traditional plant protection methods do not always provide long-term effectiveness and environmental safety, which requires the introduction of innovative approaches to creating sustainable varieties. CRISPR-Cas technology opens up new opportunities for targeted genome editing, allowing the modification or silencing of susceptibility genes and thus increasing plant resistance to fungal infections. This review presents current achievements and prospects for the application of CRISPR-Cas technology to increase the resistance of major agricultural crops to fungal diseases. The implementation of these approaches contributes to the creation of highly productive and resistant varieties, which is crucial for ensuring food security in the context of climate change.

## 1. Introduction

Plant diseases are one of the most significant threats to agriculture and global food security. Given that the current world population of 7.6 billion is expected to reach 8.6 billion by 2030, 9.8 billion by 2050, and 11.2 billion by 2100, combating crop diseases is crucial to ensuring food security and maintaining sufficient food production to meet growing global demand [1]. According to data from the Food and Agriculture Organization of the United Nations [2], diseases and pests cause 20–40% of global crop losses each year, equivalent to billions of dollars in economic losses. The economic impact of crop diseases goes far beyond direct crop losses. In countries where agriculture is the main source of income and employment, especially in developing countries, disease outbreaks cause catastrophic damage, particularly to small farmers, who often do not have access to modern plant protection products. These epiphytotics cause food prices to rise, disrupt trade chains, and provoke food shortages, exacerbating the problems of hunger and malnutrition [3]. Plant diseases also pose a serious obstacle to the implementation of the Zero Hunger program, as they cause losses of up to 40% of the global harvest each year [4], directly threatening food security and contributing to rising levels of hunger.

Diseases caused by fungal pathogens pose a serious threat, affecting major agricultural crops—cereals, legumes, and vegetables—and leading to significant crop losses and reduced product quality. Among plant diseases, fungal pathogens are considered the most destructive, causing a wide range of infections in major agricultural crops. In cereal crops, the most common fungal diseases are powdery mildew, rusts (leaf, stem, and yellow), fusarium head blight, smut, and helminthosporium root rot [5]. Legumes are often affected by fungi of the genera *Ascochyta* and *Rhizoctonia*, which cause ascochyta blight and fusarium wilt [6]. Vegetable crops suffer from a variety of fungal diseases, including powdery mildew, late blight, leaf spot, anthracnose, and various root rots [7].

Currently, the main strategy for combating plant diseases remains the use of chemical pesticides. However, the use of pesticides often has direct and indirect negative effects on the environment and other living organisms [8]. Moreover, due to the widespread and uncontrolled use of these substances, phytopathogens are gradually developing resistance to them [9]. Reducing the negative consequences of the chemicalization of agricultural systems requires the search for alternative solutions. One of the most effective and environmentally friendly approaches is the creation of disease-resistant varieties through plant breeding [10,11]. Traditional breeding, despite its past successes, faces a number of limitations: the complexity of introgression of resistance genes (*R* genes), limited genetic variability, the transfer of undesirable traits along with target traits, and the labor-intensive nature of the process [12,13]. In addition, the pace of traditional breeding does not always keep up with the rate of pathogen evolution and growing global demand for food, especially in the context of global climate change [14].

**Figure 1:**
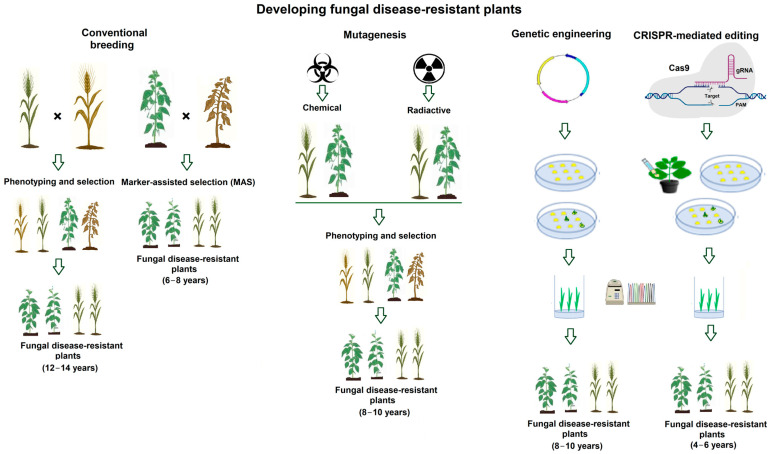
Developing plants resistant to fungal diseases. Conventional plant breeding (**left panel**); Mutagenesis (**middle panel**); and Genetic engineering and CRISPR-mediated gene editing technology (**right panel**) for improving fungal disease resistance in plants. In conventional breeding, developing fungal disease–resistant crops typically take 12–14 years (**left panel**), while marker-assisted selection (MAS) reduces this to 6–8 years (**middle panel**). Using mutagenesis or genetic engineering tools requires approximately 8–10 years (**right panel**). By contrast, CRISPR-mediated genome editing can shorten the development time to 4–6 years (**right panel**).

Alternative approaches to plant breeding, such as mutation breeding and transgenesis [15,16] are also used, but they have limitations that reduce their effectiveness and public acceptance (Figure 1).

Achieving the goal of ending hunger by 2030 requires comprehensive approaches, including the development of disease-resistant crops using methods such as clustered regularly interspaced short palindromic repeats (CRISPR)-CRISPR-associated (Cas) proteins, and the implementation of sustainable disease and pest control strategies that enable the precise, rapid, and reproducible creation of genetically improved lines [17].

The basic mechanism of genome editing using technologies such as CRISPR-Cas9, is to create double-stranded breaks (DSBs) at a specific point using a template in the genome and utilize the host non-homologous end joining (NHEJ) mechanism to create indel mutations and subsequent gene knockout. Most studies using CRISPR-Cas9 technology are based on knockout of specific genes to produce a particular beneficial phenotype. For example, knocking out susceptibility genes (*S* genes) and introducing *R* genes, and many other examples are presented in Table 1, Table 2 and Table 3. However, insertion of DNA fragments at specific oligonucleotide-directed points in the genome using CRISPR-Cas9 has yet to be completed. Fragment insertion utilizes homology-directed repair (HDR), which is a mechanism in cells to repair double-stranded DNA damage [18].

The frequency of knock-in is much lower due to the more favorable activation of the NHEJ mechanism in eukaryotic cells compared to HDR [19]. In addition, for homology-directed repair, the donor DNA fragment must be near the cutting side of the genome and the cell must be in late S and G2 phases for HDR to occur, whereas NHEJ can occur at any time other than mitosis [20]. The first genome editing by HDR in plants was carried out in *Arabidobsis thaliana*, where the kanamycin resistance gene was inserted into the ADH1 gene locus. Hereditary events were identified by PCR-based genotyping, characterized by Southern blotting, and confirmed at the sequence level with up to 8.3% efficiency [21]. One way to increase the efficiency of HDR is to stimulate this pathway. Blocking NHEJ is one way to increase the efficiency of HDR. KU70/KU80, DNA PKcs, artemis, and ligase IV are NHEJ proteins that are overexpressed during double-strand breaks [22]. Targeting these proteins can suppress NHEJ activity and increase HDR efficiency. SCR7 is a protein that targets ligase IV by binding to the DNA-binding domain of ligase IV [23]. Another way to enhance the efficiency of HDR is to stimulate its activity using the small molecule RAD51-stimulatory compound 1 (RS-1), which stabilizes RAD51, considered as an enhancer of HDR [24]. Overexpression of RAD51 showed a six-fold increase in the HDR pathway [25], and in *A. thaliana*, overexpression of RAD51 showed up to 10-fold expression [26].

Conventional sequence-specific RNA-guided nucleases (such as Cas9) induce double-strand breaks in DNA, leading to either mutations via inaccurate NHEJ repair or precise DNA sequence replacement via HDR [27]. However, as demonstrated in numerous studies, this HDR pathway, which enables precise genome editing in plants, is highly inefficient [28]. Among current CRISPR–Cas technologies capable of inducing target DNA changes in vivo, prime editors offer high versatility, specificity, and precision. Prime Editor does not require double-strand DNA breaks and enables virtually any substitution, small insertion, or small deletion in DNA within living cells. Prime editing requires, at a minimum, a programmable nickase fused to a polymerase enzyme and an extension guide RNA that identifies the target site while serving as a template for the desired genome edit [29]. Base editing is one such example. Base editing can introduce point mutations such as CG → TA, AT → GC, and CG → GC depending on type of prime editor without generating direct DSBs or requiring DNA donors [30]. Base editors have already been utilized in crops such as rice, potato, wheat, and cotton to improve starch structure, confer herbicide tolerance, and enhance other phenotypes, as demonstrated in the review by Peng et al. showing high potential for future fungal resistance [31].

The integration of traditional breeding and genetic engineering using cloned *R* genes provides a sustainable pathway to creating crop varieties with a broad spectrum of disease resistance; the introduction of such genes effectively enhances their biotic resistance [32]. CRISPR-Cas technology is based on the system’s ability to precisely target specific regions of the genome to create desired mutations (knockouts or knock-ins). This process is facilitated by a guide RNA (sgRNA), which recognizes the target region, and the Cas9 endonuclease, which cuts the DNA at the specified position. Modern genome editing platforms allow increasing plant resistance to diseases in various ways: by knocking out *S* genes, replacing alleles through homology-directed repair, introducing *R* genes, modifying the regulation of *R/S* gene expression, and through multiplex editing targeting multiple resistance and susceptibility factors simultaneously [33,34,35].

This review summarizes current and promising achievements that open up new opportunities for applying CRISPR-Cas technology to increase the resistance of major agricultural crops (cereals, legumes, and vegetables) to fungal diseases. This technology allows targeted modification or silencing of *S* genes, providing an effective and environmentally safe alternative to traditional plant protection methods. The use of CRISPR-Cas paves the way for the creation of sustainable varieties with high resistance and yield, which is key to ensuring global food security in the face of climate change.

## 2. CRISPR–Cas Genome Editing to Improve Fungal-Disease Resistance in Main Cereal Crops

### 2.1. Wheat

Wheat (*Triticum aestivum* L.) is one of the most important food crops, providing a significant portion of global grain consumption. However, its production faces growing threats related to climate change, the spread of new pathogens, and the declining effectiveness of traditional plant protection methods [36,37,38]. Previous studies have identified 33 *S* genes that act as negative regulators of resistance. It is proposed to use the CRISPR-Cas system to modify these *S* genes, which will allow the creation of mutant lines with increased resistance to biotic stress and enrich the genetic resources of wheat for further selection [39].

Fusarium head blight (FHB), caused mainly by *Fusarium graminearum*, is a serious threat to global wheat production [40]. The most studied resistance locus, *Fhb1*, first discovered in Chinese germplasm, provides partial protection against FHB. The key *S* gene associated with this locus is *TaHRC*, which encodes a histidine-rich calcium-binding protein (Table 1). Its deletion affecting the start codon (TaHRC-S) confers resistance to infection [41]. The identity of *TaHRC-R* alleles in different genotypes indicates a single origin of the resistant allele, presumably associated with the Dahongpao haplotype. For targeted editing of *TaHRC*, a gRNA delivery system based on barley mosaic virus (BSMV) was developed in the varieties ‘Bobwhite’ and ‘Everest’ [42]. Additionally, *TaHRC S* genes were successfully edited using the CRISPR-Cas9 system delivered through interspecific hybridization of wheat with corn expressing Cas9 and gRNA. Among plants regenerated from haploid embryos in vitro, mutations at the target loci were found in 15–33% of samples, demonstrating the effectiveness of this approach [43].

The mycotoxin deoxynivalenol (*DON*) is of particular importance as a key factor in the virulence of *F. graminearum*. When comparing infection with the wild type and the Δtri5 mutant, which does not produce *DON*, approximately 400 differentially expressed genes activated by the mycotoxin were identified. Among them, *TaNFXL1* was identified, whose expression level is higher in susceptible genotypes. Functional analysis using virus-induced gene silencing (VIGS) and CRISPR confirmed that *TaNFXL1* is a negative regulator of resistance, and its knockout increases plant resistance to *FHB* [44]. Thus, targeting the *TaHRC* and *TaNFXL1* genes using CRISPR-Cas is a promising strategy for increasing wheat resistance to fusarium head blight and reducing *DON* accumulation in grain.

Another necrotrophic pathogen, *Parastagonospora nodorum*, causes wheat Septoria nodorum blotch (SNB), which leads to significant yield losses and grain quality deterioration [45]. To edit the *Tsn1* and *Snn5 S* genes to *P. nodorum*, Poddar et al. developed an approach based on the use of the Cas9–ribonucleoprotein (RNP) complex, which allows for the effective selection of gRNA and editing in wheat protoplasts and immature embryos without the introduction of exogenous DNA [46]. This method provides heritable mutations without the need for transgenic plant selection, opening up new opportunities for accelerated selection of resistant varieties. In addition, *P. nodorum* protoplasts were transformed with a Cas9–sgRNA complex targeting the *Tox3* gene, which resulted in 100% editing efficiency. When co-transformed with an HDR cassette containing 1 kb homologous flanks, the frequency of homologous recombination exceeded 70%, and when microhomologies (50 bp) were used, it reached 25%. These results confirm the high accuracy and versatility of CRISPR-Cas9 technology for editing the *P. nodorum* genome [47].

Powdery mildew of wheat, caused by the obligate biotrophic fungus *Blumeria graminis* f. sp. *tritici*, is one of the most devastating diseases of the crop, causing up to 5% of global wheat yield losses annually [48]. Loss-of-function mutations in the Powdery Mildew Locus O (*MLO*) gene provide broad resistance to the disease but are often accompanied by reduced yield. Li et al. described the Tamlo-R mutant line with a 304 kb deletion in the *MLO-B1* locus, characterized by high resistance while maintaining productivity. The deletion alters the chromatin structure and activates the tonoplast transporter *TaTMT3B*, which compensates for the negative effects of *MLO* knockout [49]. Similar results were obtained when the *TaEDR1* gene was suppressed by virus-induced silencing or RNA interference (RNAi), which increased wheat resistance to powdery mildew, indicating the role of *TaEDR1* as a negative regulator of resistance. No off-target changes were detected in the *TaEDR1* mutants obtained. Such plants showed resistance to powdery mildew and did not exhibit pathogen-induced cell death [50]. Knockout of homologues of this gene in wheat (*TaMLO*) using the CRISPR-SpCas9 system led to the formation of resistance to the powdery mildew pathogen *B. tritici* [51]. Thus, editing the *MLO* and *TaEDR1* genes using CRISPR-Cas accelerates the introduction of resistance alleles into elite varieties, ensuring the creation of productive and resistant crops and providing valuable germplasm for further breeding.

Wheat rust (yellow, leaf, and stem rust) is one of the main causes of reduced yields in this crop. Yellow (*Puccinia striiformis f.* sp. *tritici*) and leaf (*Puccinia triticina* Erikss) rusts are among the most widespread and dangerous diseases of wheat and are the main factor negatively affecting wheat yield and quality, causing significant economic damage [52,53,54]. A study by Liu et al. showed that suppression of the *TaMKP1* gene using CRISPR-Cas9 increases wheat resistance to yellow rust and powdery mildew, as well as increasing yield without infection. It has been established that *TaMKP1* interacts directly with activated *TaMPK3/4/6*, and *TaMPK4* interacts with TaPAL, indicating its role as a negative modulator of MAPK signaling [55]. Wang et al. identified the *TaPsIPK1* gene, which encodes a cytoplasmic kinase-like receptor that contributes to susceptibility to *P. tritici.* The fungal effector *PsSpg1* binds to *TaPsIPK1*, enhancing its kinase activity and inducing phosphorylation of the transcription factor *TaCBF1d*. Inactivation of *TaPsIPK1* via CRISPR-Cas9 provides broad-spectrum resistance to the pathogen without negatively affecting agronomic traits, as confirmed by two years of field trials [56]. In addition, Liu et al. demonstrated that the *TaGW2* gene, known as a negative regulator of grain weight, also reduces wheat resistance to leaf rust by ubiquitinating the *TaSGT1* protein. Editing *TaGW2* using CRISPR-Cas9 simultaneously increased grain weight and resistance to leaf rust disease [57].

Recent studies show that the CRISPR-Cas system is a powerful tool for targeted editing of wheat *S* genes and creating resistant lines without compromising yield. Modification of key genes such as *Fhb1*, *TaHRC*, and *TaNFXL1* increases wheat resistance to fusarium head blight, while loss of function of *Tsn1*, *Snn5*, and *Tox3* reduces susceptibility to SNB. Mutations in the *MLO*, *TaEDR*, and *TaMKP1* genes provide broad resistance to powdery mildew, while changes in *TaMKP1*, *TaGW2*, and *TaPsIPK1* contribute to increased resistance to leaf and yellow rust, respectively. The combination of CRISPR-Cas with VIGS, protoplast and RNP editing methods, as well as interspecific hybridization, opens up new opportunities for accelerated and accurate selection. These approaches not only enable the development of resistant and high-yielding varieties, but also form the scientific basis for the precise control of wheat immunity, expanding its genetic diversity and resistance to biotic stresses.

### 2.2. Rice

Rice (*Oryza sativa* L.) is one of the world’s most important cereal crops, ranking third in terms of production volume after corn and wheat, and serving as the staple food for nearly half of the world’s population. Rice plants are affected by a variety of diseases caused by fungi, bacteria, viruses, and nematodes, which attack various parts of the plant and significantly reduce yields. Among fungal pathogens, the most dangerous and widespread are rice blast disease (*Magnaporthe oryzae*) and rice false smut (*Ustilaginoidea virens*), which affect plants at all stages of development and manifest themselves in the form of lesions on the leaves, neck, and panicle [58].

*M. oryzae*, the causative agent of rice blast disease, is one of the most destructive pathogens of rice. Unlike toxic plasmid expression of Cas9, the RNP approach provides high accuracy and efficiency, including point substitutions and multiple mutations in the *ALB1* and *RSY1* genes, and allows the production of isogenic strains that do not contain foreign DNA [59]. In rice, *OsERF922*, which encodes the AP2/ERF transcription factor, regulates plant defense. Its knockout via CRISPR-SpCas9 increased resistance to *M. oryzae* without affecting growth and yield [60]. Similarly, a mutation in *OsSEC3A*, a subunit of the exocyst, caused activation of defense pathways and dwarfism, enhancing resistance to rice blast disease [61]. Wang et al. used the Mo_tRNAArg24-gRNA-Cas9 cassette to significantly improve gene editing efficiency in *M. oryzae*. When tested on five genes (*Rei1*, *Ppg1*, *Bip1*, *Bip2*, *Dbf2*) located on different chromosomes, the Mo_tRNAArg24-gRNA-Cas9 cassette demonstrated high gene editing efficiency in *M. oryzae* (66.7–100%) without Cas9 toxicity, including two previously uncharacterized genes, with a mutation frequency of 66.7–100% without Cas9 toxicity [62].

Functional knockout of *Pi21* in the Nanjing 9108 variety [63] and co-editing of *Pita*, *Pi21*, and *ERF922* (vector pC1300-2×35S:: Cas9-gPita-gPi21-gERF922) in line L1014 resulted in high mutation frequencies (75–85%) and a significant increase in resistance to rice blast disease [64]. Multiplex editing of *TMS5*, *Pi21*, and *Xa13* created non-transgenic triple mutants *tms5/pi21/xa13* that are resistant to blast and bacterial blight, demonstrating the potential of hybrid rice breeding [65]. The CRISPR-Cas9 system targeting *Pi21* and *OsSULTR3;6* in the susceptible variety 58B achieved >90% editing efficiency and created lines with increased resistance to rice blast and bacterial leaf streak while maintaining yield [66]. Similarly, mutations in *Bsr-d1*, *Pi21*, and *ERF922* in the Longke638S line contributed to double resistance to blast and bacterial blight diseases without reducing productivity [67]. In the Dalixiang variety, *Pi21* knockout increased resistance without altering grain quality [68], and editing *Pid3* using *Agrobacterium tumefaciens* yielded nine homozygous *pid3* mutants without the Cas9 locus, demonstrating a similar effect [69].

**Table 1:** Using CRISPR-Cas technology to improve fungal disease resistance in cereals.

Pathogen	Target Gene	Delivery Method	Type of Editing	References
Wheat				
*F. graminearum*	*Fhb1*	Particle bombardment	Deletion mutation	[41]
*F. graminearum*	*TaHRC*	Particle bombardment transformation for Cas9 and viral mediated transformation for gRNA	Knockout	[42]
*F. graminearum*	*TaHRC*	*Agrobacterium*- mediated transformation	Knockout	[43]
*P. nodorum*	*Tsn1 and Snn5*	Cas9-RNP-mediated editing	Knockout	[46]
*P. nodorum*	*Tox3*	RNP complex targeting	Knockout	[47]
*B. graminis* f. sp. *tritici*	*MLO-B1*	Cas9-RNP mediated editing	Deletion	[49]
*B. graminis* f.sp. *tritici*	*TaMLO*	Particle bombardment	Knockout	[51]
*B. graminis* f. sp. *tritici*	*TaEDR1*	Particle bombardment	Knockout	[50]
*B. graminis* f. sp. *tritici* *P. striiformis* f. sp. *tritici*	*TaMKP1*	*Agrobacterium*- mediated transformation	Knockout	[55]
*P. striiformis* f. sp. *tritici*	*TaPsIPK1*	*Agrobacterium*-mediated transformation	Knockout	[56]
*P. triticina* Erikss	*TaGW2*	*Agrobacterium*-mediated transformation	Knockout	[57]
Rice				
*M. oryzae*	*ALB1 and RSY1*	Specific base pair edits	Base editing	[59]
*M. oryzae*	*OsERF922*	Protoplast transformation	Mutation	[60]
*M. oryzae*	*OsSEC3A*	Protoplast transfection	Mutation	[61]
*M. oryzae*	*Rei1*, *Ppg1*, *Bip1*, *Bip2*, *Dbf2*	Protoplast transformation	Mutation	[62]
*M. oryzae*	Pi21	*Agrobacterium*- mediated transformation	Knockout	[63]
*M. oryzae*	*Pita*, *Pi21 and ERF922*	*Agrobacterium*- mediated transformation	Knockout	[64]
*M. oryzae*	*TMS5*, *Pi21 u Xa13*	*Agrobacterium*- mediated transformation	Mutation	[65]
*M. oryzae*	*Pi21 u OsSULTR3;6*	*Agrobacterium*- mediated transformation	Knockout	[66]
*M. oryzae*	*Bsr-d1*, *Pi21 u ERF922*	*Agrobacterium*- mediated transformation	Knockout	[67]
*M. oryzae*	*Pi21*	*Agrobacterium*- mediated transformation	Knockout	[68]
*M. oryzae*	*Pid3*	*Agrobacterium*- mediated transformation	Knockout	[69]
*M. oryzae*	*Bsr-d1*, *Perox3*	*Agrobacterium*- mediated transformation	Knockout	[70]
*M. oryzae*, *U. virens*	*MoATG3*, *MoATG7 u UvPal1*	*Agrobacterium*- mediated transformation	Knockout	[71]
*M. oryzae*	*rBE5*	*Agrobacterium*- mediated transformation	Base editing	[72]

The *bsr-d1* allele, encoding a transcription factor from the Digu variety, provides broad resistance to *M. oryzae* by suppressing H_2_O_2_ degradation. Knocking out *Bsr-d1* induced redox status, amino acid and fatty acid metabolism, and salicylic acid signaling pathways, and identified a new target gene, *Perox3*, which reduces resistance when overexpressed [70]. Recently, Zhang et al. developed dCas9-based CRISPR interference (CRISPRi) to suppress genes of the fungal pathogens *M. oryzae* and *U. virens*. The use of gRNAs targeting the *MoATG3*, *MoATG7*, and *UvPal1* promoters showed that a single gRNA can effectively reduce expression and induce expected phenotypes, making CRISPRi a powerful tool for functional genomics of pathogens [71].

In addition to the knockout strategy, a promising approach is precise base editing, which allows for the correction of defective alleles of *R* genes. Precise base editing also opens up possibilities for the correction of *R* genes. For example, Ren et al. used the cytidine editor rBE5 to correct a single nucleotide substitution (G > A) in the recessive allele *pi-d2*, restoring resistance to rice blast disease [72].

Recent studies show that CRISPR-Cas technology has significantly accelerated the development of disease-resistant rice varieties. Knockout of key *S* genes (*Pi21*, *Bsr-d1*, *OsERF922*, *Pid3*, etc.) has provided effective protection against *M. oryzae* without reducing yield and grain quality. The development of improved systems, such as plasmid-free RNP complexes and Mo_tRNAArg24-gRNA-Cas9-type cassettes, has increased the accuracy and efficiency of editing, while the emergence of CRISPRi and base editing has opened up opportunities for fine-tuning the expression and restoring the functionality of *R* genes. These advances form the basis for precision breeding of rice and the creation of sustainable, productive, and environmentally safe varieties.

### 2.3. Barley

Barley (*Hordeum vulgare* L.) is one of the most important cereal crops, widely used in brewing, feed production, and the food industry [73]. Barley production is limited by the impact of various pathogens that cause fungal, bacterial, and viral diseases. The most common diseases include dwarf rust, powdery mildew, fusarium, and various forms of smut [74].

The use of CRISPR-Cas technology opens up broad prospects for increasing barley’s resistance to disease through targeted editing of genes involved in the formation of the plant’s natural defense mechanisms against pathogens.

Gene replacement or inactivation can be achieved by combining Cas9/sgRNA RNP with a PCR-amplified selective marker containing short homologous flanks of the barley target gene for fungal transformation. This approach eliminates the need for labor-intensive vector construction procedures and ensures high precision of targeted knockout [75]. Colonization of plants by fungal pathogens is a complex multistep process involving numerous genes in both the host plant and the pathogen. In recent years, particular attention has been paid to the study of *S* genes, which are negative regulators of plant defense mechanisms. One of the best-known examples is the *MLO* locus, first described in barley as a susceptibility factor to powdery mildew [76].

Research on editing the barley genome using CRISPR-Cas technology is actively developing, but most of it focuses on improving key agronomic traits—yield, drought resistance, starch content, and disease resistance. Cas technology is actively developing, but most of it is focused on improving key agronomic traits—yield, drought tolerance, starch and protein content—as well as creating resistance to viral infections, including barley yellow dwarf virus (BYDV) and mosaic virus. At the same time, there is still not much work being done on making barley more resistant to fungal pathogens like rust, powdery mildew, fusarium, and smut. This is due to the complexity of molecular interactions in the plant-pathogen system and the insufficient study of *S* genes in barley. Nevertheless, given the successful application of CRISPR-Cas technology for editing similar *S* genes in wheat and rice, the use of this approach to target homologous genes in barley is a promising direction for increasing the crop’s resistance to fungal diseases and expanding its genetic potential.

## 3. CRISPR-Cas Genome Editing for Improvement Fungal Disease Resistance of Legumes

Soybean (*Glycine max* L.) is one of the most economically important leguminous crops, grown and consumed worldwide as a source of protein and oil for animal feed and human nutrition [77]. However, global soybean production is seriously threatened by many diseases, one of which is powdery mildew caused by the obligate biotrophic fungus *Erysiphe diffusa* [78].

Bui et al. developed CRISPR-Cas9 constructs with dual sgRNA, which were successfully transferred to the Vietnamese soybean variety DT26 through transformation using *A. tumefaciens* (Table 2). Various mutant forms of the *GmMLO* genes, including biallelic, chimeric, and homozygous, were detected in the T0 generation. The inheritance and segregation of CRISPR-Cas9-induced mutations were confirmed and validated in the T1 and T2 generations. From the six GmMLO genes in the soybean genome, the authors obtained triple knockout mutants *Gmmlo02*/*Gmmlo19*/*Gmmlo23* and quadruple knockout mutants *Gmmlo02*/*Gmmlo19*/*Gmmlo20*/*Gmmlo23* in the T2 generation. When infected with *E. diffusa*, a fungus that causes powdery mildew in soybeans, all mutant plants showed increased resistance to the pathogen, especially the quadruple mutant. The degree of powdery mildew infection in mutant soybeans was reduced by 36.4% compared to wild-type plants. In addition, no pleiotropic effects on the growth and development of soybeans were observed in CRISPR-Cas9 mutants under greenhouse conditions [79].

In another study, the CRISPR-Cas9 system was successfully introduced into the oomycete pathogen *Phytophthora sojae*, enabling rapid and efficient genome editing. Using the *RXLR Avr4/6* effector gene as a target, the researchers observed that in the absence of a homologous template, DSBs induced by Cas9 were repaired by NHEJ, which mainly resulted in short insertions or deletions (indels). The *Avr4/6* gene in *P. sojae* encodes a single RXLR-dEER effector protein of 123 amino acids, which determines avirulence in soybean plants carrying the *Rps4* or *Rps6* resistance genes. Initially thought to be two separate genes, *Avr4* and *Avr6* turned out to be a single locus, *Avr4/6*, located in a 24-kilobyte region of the genome [80].

**Table 2:** Using CRISPR-Cas technology to improve fungal-disease resistance in legumes.

Pathogen	Target Gene	Delivery Method	Type of Editing	References
Soybean				
*E. diffusa*	*GmMLO02*, *GmMLO19*, *GmMLO20*, *GmMLO23*	*Agrobacterium*-mediated transformation	Knockout	[79]
*P. sojae*	*RXLR Avr4/6*	(PEG)-mediated protoplast transformations	Knockout	[81]

The authors note that most mutants were homozygous, presumably as a result of gene conversion caused by Cas9-mediated cleavage of non-mutant alleles. In the presence of donor DNA, HDR was observed, which led to the replacement of *Avr4/6* with the NPT II genome. By testing the specific virulence of several NHEJ mutants and HDR-mediated gene replacements in soybean, we confirmed the contribution of *Avr4/6* to recognition by the soybean R gene loci *Rps4* and *Rps6*, but also found an additional contribution of these two loci to resistance [81].

These studies demonstrate the effectiveness of CRISPR-Cas9 technology in enhancing soybean resistance to powdery mildew and investigating the mechanisms of *P. sojae* pathogenicity. Targeted editing of host *S* genes (*GmMLO*) and pathogen effectors (*Avr4/6*) provides valuable information and practical tools for creating disease-resistant soybean varieties.

## 4. CRISPR-Cas Genome Editing for Improvement Fungal Disease Resistance of Vegetables

### 4.1. Tomato

Tomato (*Solanum lycopersicum* L.) is one of the world’s most important vegetable crops. According to data from the Food and Agriculture Organization of the United Nations (FAO) as of September 2025, it is cultivated on an area of 5.41 million hectares, with a total production of 192 million tons and an average yield of 35.5 tons per hectare. Pathogens establish compatible interactions by exploiting host factors encoded by plant *S* genes. Data indicate that inactivating plant *S* genes can confer broad-spectrum and persistent resistance [82].

Powdery mildew is a widespread fungal disease that causes significant economic damage to crops such as wheat, barley, and tomatoes, mainly in temperate climates [83]. It has been found that plants resistant to powdery mildew carry recessively inherited mutations with loss of function in the *MLO* gene [84]. In *MLO* mutant plants, powdery mildew pathogenicity is arrested at the stage of cell wall penetration and host cell invasion. As a result, fungal sporophytes cannot form haustoria in host cells, and fungal colonies do not develop. Thus, this gene has become the subject of interest in many studies on genome editing to obtain resistance to fungal diseases [85].

One of the first studies on the use of the CRISPR-Cas system with a knocked-out *MLO* gene for tomatoes was conducted in 2017 by the team of Nekrasov et al. In the study, the authors report the creation of the Tomelo tomato variety, which does not contain transgenic elements and is resistant to the fungal pathogen powdery mildew, using CRISPR-Cas9 technology in less than ten months (Table 3). The study also performed whole-genome sequencing to show that the resulting mutant line does not contain any foreign DNA sequences, but only has a deletion that is indistinguishable from natural mutations. The group also presented evidence that CRISPR-Cas9 is a highly accurate tool, as no off-target mutations were found in Tomelo. Thus, it was shown that mutations can be easily introduced into elite or locally adapted tomato varieties in less than a year with relatively minimal effort and investment [86].

**Table 3:** Using CRISPR-Cas technology to improve fungal disease resistance in vegetables.

Pathogen	Target Gene	Delivery Method	Type of Editing	References
Tomato				
*Oidium neolycopersici*	*SlMlo1*	*Agrobacterium*- mediated transformation	Knockout	[86]
*O. neolycopersici*	*SlMlo1*, *SlPelo*	*Agrobacterium*- mediated transformation	Knockout	[87]
*O. neolycopersici*	*PMR4*	*Agrobacterium*- mediated transformation	Knockout	[88]
*O. neolycopersici*	*SlDND1*	*Agrobacterium*- mediated transformation	Knockout	[89]
*Phytophthora infestans*	*SlPMR4*	*Agrobacterium*- mediated transformation	Knockout	[90]
*P. infestans*	*miR482b*, *miR482c*	*Agrobacterium*- mediated transformation	Knockout	[91]
*Phytophthora capsici*	*SlDMR6-1*	*Agrobacterium*- mediated transformation	Knockout	[92]
*Fusarium oxysporum*	*XSP10*, *SlSAMT*	*Agrobacterium*- mediated transformation	Knockout	[93]
*F. oxysporum*	*SlPUB21*, *SlPUB17/SlPUB21*	*Agrobacterium*- mediated transformation	Knockout	[94]
*Botrytis cinerea*	*SlPLC2*	*Agrobacterium*- mediated transformation	Knockout	[95]
*B. cinerea*	*SlPG2a*, *SlPL*	*Agrobacterium*- mediated transformation	Knockout	[96]
*Verticillium dahliae*	*SlWAT*	*Agrobacterium*- mediated transformation	Knockout	[97]
Pepper				
*Leveillula taurica*	*CaMLO2*	RNP	Knockout	[98]
*L. taurica*	*CaMLO2*	RNP	Knockout	[99]
*Colletotrichum truncatum*	*CaERF28*	*Agrobacterium*-mediated transformation	Knockout	[100]
Eggplant				
*P. infestans*, *P. capsici*	*SmDMR6*	*Agrobacterium*-mediated transformation	Knockout	[101]

Another group of researchers also obtained knockout lines for the *SlMlo1* gene, demonstrating complete resistance to powdery mildew fungus. The results demonstrate the effectiveness of the CRISPR-Cas9 system for introducing targeted mutagenesis to rapidly develop pathogen-resistant tomato varieties, which is consistent with the results of a previous study. The study used the CRISPR-Cas9 system to obtain *SlMlo1* knockout mutants by transforming tomatoes with Agrobacterium in the elite tomato line BN-86. The authors succeeded in creating *SlMlo1* knockout mutant lines that demonstrated complete resistance to the fungal disease powdery mildew [87].

Another gene discovered in *A. thaliana* mutants resistant to powdery mildew through screening was named “powdery mildew resistance” (*PMR*). These mutants demonstrated resistance to *Golovinomyces cichoracearum* and *Golovinomyces orontii* [102]. Santillán Martínez et al. used the ortholog of the *PMR* gene in tomato to test the resistance of new mutant lines to *O. neolycopersici*, a powdery mildew pathogen. They used a CRISPR-Cas9 construct containing four single-guide RNAs (sgRNAs) targeting the tomato *PMR4* gene to increase the likelihood of large deletions in mutants. After selection based on PCR and sequencing of transformants, five different mutational events were identified, including deletions ranging from 4 to 900 bp, a 1 bp insertion, and an 892 bp inversion. All of these mutants showed reduced susceptibility to *O. neolycopersici* based on visual assessment of disease symptoms and quantitative assessment of relative fungal biomass [88].

Previous studies on potatoes have shown that disruption of *DND1* (Defense No Death 1) function increases plant resistance to various pathogens, such as powdery mildew (PM) *O. neolycopersici*, but this comes at the cost of negative effects on overall plant health and viability, such as the expression of a severe dwarf phenotype, autonecrosis, and reduced male fertility [103].

To explore the possibility of minimizing the negative effects of the *DND1* mutation while simultaneously increasing disease resistance, Li et al. developed a CRISPR-Cas9 construct with four single-target RNAs targeting three exons of *SlDND1* (Solyc02g088560.4.1), which was introduced into the tomato cultivar Moneymaker (MM) via transformation with *A. tumefaciens*. Three T1 lines (named E1, E3, and E4) were crossed with MM and then self-pollinated to produce TF2 families. All TF2 plants in the homozygous *dnd1/dnd1* state showed a reduction in PM symptoms compared to heterozygous (*DND1/dnd1*) and wild-type (*DND1/DND1*) plants. Two complete knockout mutants (E1 and E4), encoding truncated *DND1* proteins, showed clear phenotypes of dwarfism and autonecrosis, while the E3 mutant, carrying a deletion of 3 amino acids, showed normal height growth with fewer autonecrotic spots. Analysis of the three-dimensional structures of both the reference and mutant proteins revealed significant conformational changes in the protein derived from E3, which could potentially affect its function. The *dnd1*/*dnd1* TF2 (TV181848-9, E3) was subjected to whole-genome sequencing using Illumina technology, which confirmed the absence of off-target mutations in selected regions of the genome. In addition, no traces of the Cas9 gene were found, indicating its elimination by segregation [89].

Another group, Li et al., also used the *SlPMR4* gene in their study. A CRISPR–Cas9 vector containing four single-stranded RNA guides (sgRNA: sgRNA1, sgRNA6, sgRNA7, and sgRNA8) targeting the same number of *SlPMR4* regions, was introduced by transformation using *A. tumefaciens* into two widely grown Italian tomato varieties: San Marzano (SM) and Oxheart (OX). Thirty-five plants (26 SM and 9 OX) were selected and analyzed to identify CRISPR-Cas9-induced mutations. Different sgRNAs caused mutations with a frequency ranging from 22.1 to 100% and, depending on the type, precise insertions (sgRNA6) or deletions (sgRNA7, sgRNA1, and sgRNA8). sgRNA7 induced a −7 bp deletion in seven SM genotypes in the homozygous state, while sgRNA8 resulted in fifteen SM genotypes with a biallelic mutation (−7 bp and −2 bp). Selected edited lines were inoculated with *P. infestans*, and four of them, completely knocked out at the *PMR4* locus, showed a reduction in disease symptoms (decrease in susceptibility from 55 to 80%) compared to control plants. Four SM lines were sequenced using Illumina whole-genome sequencing for deeper characterization without showing any signs of mutations in candidate off-target regions. Results demonstrated for the first time a reduction in susceptibility to *P. infestans* in pmr4 tomato mutants, confirming the role of *PMR4* knockout in providing broad-spectrum protection against pathogens [90].

MicroRNAs (miRNAs) are small non-coding RNAs 20–24 nucleotides (n.p.) in length that are processed from *MIRNA* genes transcribed by RNA polymerase II. In plants, the primary transcript (pri-miRNA) of the MIRNA gene is processed by the DICER-LIKE1 (*DCL1*) enzyme, similar to RNase III, into a hairpin structure, a miRNA precursor or pre-miRNA, which is further cleaved by *DCL1* to form a miRNA/miRNA* duplex from the stem region of the hairpin. The double-stranded miRNA/miRNA* is assembled into a RNA-induced silencing complex (*RISC*) [104]. miR482 is a conserved and extensive family of microRNAs that plays a key role in regulating plant defense mechanisms by targeting transcripts with leucine-rich nucleotide-binding site (*NBS-LRR*) motifs. *NBS-LRR* genes are involved in plant immunity by mediating the recognition of pathogen effectors [105]. Hong et al. 2021 [91] obtained two transgenic plants with simultaneous suppression of *miR482b* and *miR482c* and one transgenic line with suppression of only *miR482b*. Compared to wild-type plants, disease symptoms in the three transgenic plants were reduced upon infection, accompanied by increased expression of their common target genes with nucleotide binding sites and leucine-rich repeats and decreased levels of reactive oxygen species. In addition, simultaneous suppression of *miR482b* and *miR482c* was more effective than suppression of only *miR482b* in tomatoes. It was also found that knockout of *miR482b* and *miR482c* can cause disruption of the expression of other miRNAs, indicating cross-regulation between miRNAs. The authors’ study demonstrated that simultaneous editing of *miR482b* and *miR482c* using CRISPR-Cas9 is an effective strategy for creating pathogen-resistant tomatoes, and that cross-regulation between miRNAs may reveal a new mechanism of interaction between tomatoes and *P. infestans* [91].

It has been demonstrated that inactivation of a single gene, called Downy Mildew Resistance 6 (*DMR6*), confers resistance to several pathogens in *A. thaliana* [106]. This gene is specifically activated during pathogen infection, and mutations in the *dmr6* gene lead to increased salicylic acid levels. Using the CRISPR-Cas9 system, Thomazella et al. created tomato plants with small deletions in the *SlDMR6-1* gene, which lead to gene inactivation. The authors showed that the resulting mutants have no significant adverse effects on growth and development under greenhouse conditions and demonstrate resistance to various pathogens, including *P. syringae*, *P. capsici*, and *Xanthomonas* spp. [92].

Xylem sap protein 10 (*XSP10*) and salicylic acid methyltransferase (*SlSAMT*) are two putative negative regulatory genes associated with Fusarium wilt in tomatoes. Tomato tolerance to Fusarium wilt can be developed by targeting these *S* genes. *XSP10* is a non-specific lipid transfer protein (LTP) with a molecular weight of 10 kDa, containing a motif of 8 cysteine residues that forms intramolecular disulfide bonds in tomatoes. According to research, the *XSP10* gene acts as a compatibility factor for Fol, enhancing Fol colonization in the roots of tomato plants and promoting the development of disease symptoms in plants. In addition, it transports essential lipids from the plasma membrane to pathogens, increasing susceptibility to disease and its progression [107]. *SAMT* enzymes regulate salicylic acid (SA) homeostasis in plants by catalyzing the conversion of SA to methyl salicylate (MeSA) with S-adenosyl-L-methionine (SAM) as a methyl donor. The conversion of endogenous SA to MeSA reduces the host’s defense against multiple pathogen attacks [108]. *F. oxysporum* f. sp. *lycopersici* is a fungal pathogen that attacks plant roots and ranks fifth among the most destructive fungal infections of tomatoes [109]. The fungus’s hyphae penetrate and colonize the apoplastic spaces, surrounding the stele and blocking the xylem vessels, which leads to stunted growth, leaf chlorosis, progressive wilting, and cell death [110]. In a study conducted by Debbarma et al. two *S* genes (*XSP10* and *SAMT*) were analyzed using CRISPR-Cas9 editing of one gene (*XSP10* and *SlSAMT* separately) and two genes (*XSP10* and *SlSAMT* simultaneously). Before proceeding to the creation of stable lines, the efficiency of sgRNA-Cas9 complex editing was first verified by transforming individual protoplast cells. Lines with CRISPR editing of the double genes XSP10 and *SlSAMT* in the GE1 generation showed strong phenotypic tolerance to Fusarium wilt disease compared to lines with single gene editing. Overall, genetics studies in transient and stable tomato lines showed that *XSP10* and *SlSAMT* function together as negative regulators, providing genetic tolerance to Fusarium wilt disease [93]. In earlier work by Gaona et al. the group screened a Micro-Tom EMS tomato population and found a mutant that showed reduced susceptibility to both necrotrophic fungi. The authors reported a mutation in the tomato *PUB17* gene as the cause of reduced susceptibility in this mutant [94]. The role of another gene in this family, *PUB21*, as a susceptibility factor to both necrotrophic fungi was confirmed in transformants with RNAi suppression and CRISPR mutations. The results show that *SlPUB21* plays a key role in the susceptibility of tomato plants to the necrotrophic pathogens *B. cinerea* and *Alternaria solani*. The SlPUB21 mutation provides a broad spectrum of resistance to these pathogens without causing serious pleiotropic effects. The double mutant *pub17*/*pub21* in tomatoes showed a higher level of resistance, but also exhibited reduced plant size [111].

Tomato plants with CRISPR-Cas9-induced Phospholipase C2 knockouts are more resistant to *B. cinerea* than wild-type plants. Perk et al. obtained *SlPLC2* knockout tomato lines with reduced ROS (reactive oxygen species) production upon exposure to *B. cinerea.* Since this fungus requires ROS-induced cell death for reproduction, *SlPLC2* knockout plants showed increased resistance with fewer necrotic areas and reduced pathogen reproduction. Thus, using CRISPR-Cas9 genome editing technology, we obtained tomato lines with *SlPLC2* loss of function that were more resistant to *B. cinerea*. Phosphoinositide-specific phospholipase C (*PI-PLC*) is a signaling enzyme that hydrolyzes membrane phosphoinositides to form lipids and lipid derivatives that act as secondary messengers. In plants, *PI-PLCs* are involved in various physiological processes, including immunity. It has previously been shown that tomato *SlPLC2* is involved in susceptibility to the necrotrophic fungus *B. cinerea* [95].

Ortega-Salazar et al. simultaneously knocked out two pectin-degrading enzymes, polygalacturonase (*SlPG2a*) and pectate lyase (*SlPL*), which play a key role in softening tomato fruits. It was found that *SlPG2a* and *SlPL* enzymes act additively, significantly affecting fruit firmness and shelf life, with double CRISPR knockout (CRISPR PGPL line) outperforming wild-type fruits. In addition, compared to wild-type fruits, CRISPR double knockout (PGPL) fruits showed improved or unchanged quality indicators such as sugar-to-acid ratio, aromatic volatiles, and skin color [96]. Fruit with a single CRISPR PL gene knockout is significantly less susceptible to fungal infections caused by *B. cinerea* compared to WT fruit [112]. The authors inoculated fruits of all tomato lines obtained in the study with *B. cinerea* and monitored disease development throughout the post-harvest storage period, which showed a more resistant phenotype in CRISPR PL and CRISPR PGPL line compared to the wild type [96].

The Walls Are Thin 1 (*WAT1*) gene is a disease susceptibility gene first discovered in Arabidopsis and cotton that exhibits susceptibility to *V. dahliae*, a particularly well-known vascular wilt pathogen [113]. WAT1 encodes an auxin transporter localized in the tonoplast, but its exact role in so-called “vascular immunity” is not yet clear [114]. Hanika et al. identified the tomato WAT1 homolog Solyc04g080940 (*SlWAT1*) in their study. Temporary and stable suppression of *SlWAT1* based on VIGS and RNAi, respectively, did not result in a permanent reduction in tomato susceptibility to *V. dahliae*. However, CRISPR-Cas9 tomato mutant lines carrying targeted deletions in *SlWAT1* showed significantly increased resistance to *V. dahliae*, as well as to *Verticillium alabastrum* and *F. oxysporum* f. sp. *lycopersici*. Thus, the knockout of the tomato *WAT1* gene resulted in a broad spectrum of resistance to various vascular pathogens of tomato [97].

The application of CRISPR-Cas technology to tomatoes has demonstrated impressive success in increasing resistance to major fungal pathogens such as *O. neolycopersici*, *P. infestans*, *F. oxysporum*, *B. cinerea* and *V. dahliae*. Through precise editing of susceptibility and defense genes, researchers have created lines with stable disease resistance, increased yield, and no significant growth abnormalities. These achievements demonstrate that CRISPR/Cas is a powerful and sustainable tool for breeding a new generation of tomato varieties that are resistant to fungal diseases.

### 4.2. Pepper

Pepper (*Capsicum annuum* L.) is a widely cultivated and important vegetable crop due to its high nutritional value, unique vitamin content, and diverse mineral composition [115]. However, it is susceptible to climate-driven spread of pathogens and vectors [116]. Climate change has exacerbated the problem of powdery mildew, a disease that hinders pepper growth and yield by disrupting photosynthesis and hormone production. Wind and water contribute to the spread of powdery mildew infection [117].

Park et al. used the well-known *MLO* gene for knockout in *C. annuum* using previously created guide RNAs targeting the *CaMLO2* gene, using RNP for protoplast transformation. This study confirmed the reliability of protoplast isolation and the effectiveness of Cas9/CaMLO2sgRNA1 as a gene editing tool without potential side effects in six commercial hot pepper varieties. Despite the genetic diversity of the six pepper varieties, the study of the genetic structure of *CaMLO2* homologs showed that this approach is consistent and reliable for targeting and modifying the gene [98].

Another group had previously demonstrated that pepper protoplasts derived from leaves or callus grown in soil are a useful system for screening effective guide RNAs for CRISPR-Cas9 or CRISPR-Cas12a (Cpf1). This study shows that CRISPR-Cas9 or Cpf1 were delivered as CRISPR/RNP complexes from purified endonucleases mixed with engineered single-guide RNA that can edit the target gene *CaMLO2* in two pepper varieties with sequenced complete genomes, *C. annuum* ‘CM334’ and *C. annuum* ‘Dempsey’. The engineered guide RNAs (sgRNA for Cas9 or crRNA for Cpf1) are retained for *CaMLO2* in both CM334 and Dempsey and cleave *CaMLO2* in vitro. CRISPR-Cas9- or/Cpf1-RNP complexes were transfected into freshly isolated protoplasts of CM334 hot pepper and Dempsey sweet pepper using PEG-mediated delivery. Targeted deep sequencing analysis showed that the target gene *CaMLO2* was differentially edited in both varieties depending on the CRISPR/RNP applied [99].

Another gene, also a S gene, *ERF28*, was used for knockout in pepper. This gene was selected by researchers as a gene that is overexpressed during anthracnose infection [118]. Anthracnose, caused by *Colletotrichum* species, is a major disease of chili peppers leading to significant yield losses before and after harvest in tropical and subtropical regions of the world [119]. Mishra et al. developed a construct with single expression of Cas9, sgRNA driven by the Pol II promoter to modify the *CaERF28* gene in the susceptible chili pepper genotype Arka Lohit. Of the 62 T0 transgenic plants, 45 mutant lines induced by *CaERF28* (72.5%) were identified. In addition, simultaneous exposure to multiple sites within *CaERF28* showed increased mutation efficiency (85.7%). Five homozygous mutants showed increased resistance to anthracnose compared to the wild type, as evidenced by a reduction in spore and fungal growth, as well as induced expression of defense-related genes. In addition, the authors showed that all mutant chili pepper plants were morphologically normal and agronomically similar to the wild type, suggesting that modification of the *CaERF28* locus does not interfere with plant development [100].

CRISPR-Cas technology has demonstrated high potential for improving disease resistance in peppers (*C. annuum* L.). Successful knockout of key *S* genes, such as *CaMLO2* and *CaERF28*, has demonstrated increased resistance to major fungal pathogens, such as powdery mildew and anthracnose, without negatively affecting plant growth or yield. These achievements demonstrate the effectiveness and precision of CRISPR/Cas as a tool for creating disease-resistant pepper varieties that contribute to sustainable crop production in a changing climate.

### 4.3. Eggplant

According to the FAO, eggplant (*Solanum melongena* L.) is the most important berry crop of the nightshade family after tomatoes, with a production volume of 60 million tons. Since eggplant is a vegetable that contributes to nutrition in large areas of the world, it is extremely important to develop new strategies to increase its disease resistance, and as a result, increase its production in order to cope with the rapid growth of the world’s population and the effects of climate change [120].

Among the *S* genes, *DMR6* encodes an enzyme involved in the degradation of salicylic acid (SA), and its inactivation in other Solanaceae species has been shown to increase SA levels and confer tolerance to a wide range of pathogens, as described earlier. Ferrero et al. identified two orthologs of this gene in the eggplant genome, namely *SmDMR6–1* and *SmDMR6–2*, for increased resistance to the oomycete pathogens *P. capsici* and *P. infestans*. In the Black Beauty variety, only the expression of *SmDMR6–1* was significantly increased upon infection with two oomycetes, *P. infestans* and *P. capsici*, indicating its involvement in regulating the plant’s response to biotic stresses. The authors demonstrated the knockout of the *SmDMR6–1* gene using CRISPR-Cas9 technology in eggplants. Regenerated T0 plants were screened using Sanger sequencing, one of them was selected and self-pollinated to obtain T1 and then T2 plants. The mutant lines were tested for pathogenicity, which revealed increased tolerance to infection by *P. infestans* and *P. capsici* compared to unedited plants [101].

Knockout of the *SmDMR6–1* gene in eggplant (*S. melongena* L.) using CRISPR-Cas9 successfully increased resistance to late blight, a major oomycete pathogen affecting crop yield. This demonstrates the potential of genome editing to enhance eggplant disease resistance without compromising plant growth. Such achievements represent an effective strategy for creating sustainable varieties that contribute to sustainable production and global food security in the context of climate change.

## 5. Challenges, Future Perspectives and Conclusions

In terms of an agronomic perspective, CRISPR-edited vegetables offer the potential to enhance yield stability and improve productivity by reducing losses caused by biotic stresses such as viral, fungal, and bacterial pathogens [121,122,123]. These improvements can reduce reliance on chemical pesticides, benefiting both environmental conservation and sustainable agricultural practices [34]. However, a significant agronomic concern is the potential for multifactorial effects (unintended negative side effects on plant physiology) [124,125]. For example, inactivation of the *TaMLO* gene in wheat conferred powdery mildew resistance but simultaneously caused leaf yellowing, a prominent sign of physiological stress [126]. Similarly, a *CYP71A1* gene mutation in rice enhanced resistance to whiteflies and rice stem borers but resulted in reduced grain yield in field trials [127]. Therefore, the long-term agronomic success of these varieties depends on confirming that the edited traits maintain fitness and yield under diverse field conditions through multi-environmental trials, as stated in the review [128].

Ecologically, the introduction of CRISPR-edited crops has the potential to contribute to sustainable agriculture by reducing the need for synthetic pesticides, protecting non-target organisms, and conserving biodiversity. While pesticides have historically been used to control diseases efficiently, their use must be significantly reduced considering their adverse effects on biodiversity. Plants with natural resistance to pathogens can contribute to pesticide reduction and yield protection [129]. However, the long-term ecological impacts are not fully understood. The persistence of resistance is a major concern. Pathogens may evolve to overcome resistance mechanisms, particularly when resistance relies on the inactivation of a single susceptibility gene. Furthermore, the potential for gene flow from edited crops to wild relatives remains a consideration for ecosystem integrity, though the probability is lower with non- genetically modified organisms (GMOs) editing [130]. Broader ecological impacts also depend on agricultural practices. Implementing crop rotation, reducing monocropping, and combining with integrated pest management strategies can enhance the persistence of resistance. Ultimately, ecological benefits depend on responsible introduction and monitoring to prevent unintended consequences such as the emergence of resistant pathogen strains or disruption of local ecosystems [131,132,133].

Recent studies have clearly shown that CRISPR/Cas technology has become a key tool for precision genome editing and creating fungal disease-resistant varieties of major crops. The application of this approach has made it possible to successfully modify *S* genes in wheat, rice, barley, soybeans, tomatoes, peppers, and eggplants, providing resistance to fungal pathogens such as *F. graminearum*, *F. oxysporum*, *P. nodorum*, *B. graminis f.* sp. *tritici*, *P. striiformis f.* sp. *tritici*, *P. triticina Erikss*, *M. oryzae*, *E. diffusa*, *P. sojae*, *O. neolycopersici*, *P. infestans*, *P. capsici*, *B. cinerea*, *V. dahliae*, *L. taurica* and *C. truncatum*. CRISPR-Cas technology provides high accuracy, efficiency, and reproducibility without affecting plant growth and yield, making it a powerful and environmentally safe tool for sustainable agriculture. Further development of areas related to S- and R-gene editing, the introduction of plasmid-free systems, multiplex editing, and CRISPRi regulatory platforms will open up new opportunities for accelerated breeding of crops with complex disease resistance and adaptation to changing climatic conditions.

Currently, the fastest and most reliable way to obtain genetically modified plants with the desired disease resistance phenotype is to use CRISPR-Cas technology. There are many studies demonstrating the high potential of CRISPR-Cas in obtaining new varieties in just 10 months [86]. CRISPR-Cas9 has been successfully used to combat various fungal diseases in many agricultural crops, demonstrating its potential to accelerate research in biotechnology and agronomy. CRISPR-Cas technology is mainly used for gene knockout in loss-of-function studies [134]. Given the large number of *S* genes and their conservative nature, it allows the creation of resistant varieties for a wide range of diseases, as well as the discovery of new *S* genes from one crop and their transfer to another. For example, *MLO* genes, originally discovered in *A. thaliana*, and the ortholog of this gene in tomatoes have demonstrated resistance to powdery mildew [135]. The same has happened with the PMR gene, which was also originally discovered in Arabidopsis and is used in many different crops to increase their resistance [102].

Beyond gene knockouts, CRISPR-Cas possesses diverse variants such as base editing and transcription activation/inhibition, RNAi and more [136,137]. However, despite its broad capabilities, the use of other types of CRISPR in fungal disease resistance remains quite limited. Most of the research focuses on susceptibility gene knockouts. Other editing approaches either constitute a relatively small minority of studies or are entirely dedicated to basic research [138]. Gene editing tools capable of manipulating multiple targets are extremely useful. Simultaneous expression of Cas9 and multiple gRNAs enables multiplex gene editing, where each targets a different site [139]. The tRNA–gRNA architecture has been demonstrated to efficiently produce multiple gRNAs from a single synthetic gene. This approach enables precise cleavage of the transcript in vivo by the endogenous RNases RNase P and RNase Z [140]. All sgRNAs can be organized into a tandem sequence and expressed as a single transcript from a single promoter. This single transcript undergoes post-transcriptional processing to yield individual sgRNAs [141]. The tRNA and Csy4 system offers advantages over conventional Pol III promoter-driven sgRNA expression methods due to its shorter cassette sequence and higher editing efficiency [142]. Current genome editing strategies emphasize knockout approaches, but the DSB generation capability of the CRISPR/Cas9 system provides the foundation for HDR, which is essential for precise gene insertion. However, its application remains limited [28]. Gene knock-in studies represent another avenue for further research. This technique involves inserting specific DNA sequences into precise locations via the HDR pathway. This process requires conditions such as the target sequence presented as donor DNA and molecules that trigger HDR efficacy. Non-pathogenic factors (Avr) and effector proteins are secreted by plant pathogens into plant cells during initial infection. These effectors suppress plant defense mechanisms, facilitating pathogen establishment. However, plants have evolved defense mechanisms by acquiring resistance proteins encoded by host *R* genes, which confer marked resistance against specific pathogen strains [143]. For example, *R* genes such as *RPS5* (resistance to *Pseudomonas syringae*) and *Avr* gene *AVRPphB* can enhance resistance to fungal diseases when introduced into host organisms [144]. Even when already present, these genes can be overexpressed using transcription-regulating Cas proteins.

The most widespread method of utilizing the Cas9 cassette in plants is *Agrobacterium*-mediated transformation using a plasmid containing the Cas9 protein and the sgRNA sequence [145]. After transformation, Agrobacterium inserts the CRISPR-Cas9 cassette into the plant genome, but this can lead to various undesirable consequences such as permanent expression of the Cas9 protein or Cas9 protein off-target effects [146]. In addition, the presence of the Cas9 gene in the plant genome raises ethical concerns.

The use of CRISPR/Cas genome editing technology in vegetable crops opens up prospects for increasing yields, stress resistance, and improving product quality. However, the commercialization of such crops is subject to a number of regulatory, ethical, and consumer challenges. The main difficulties are related to the lack of uniform international rules: in some countries, gene-edited plants are equated with GMOs, while in others they are exempt from these requirements if they do not contain foreign DNA. For example, the EU and New Zealand [147,148,149,150] apply strict regulations, while the US, Argentina, Brazil, Chile, and Colombia only approve products without exogenous DNA [151,152,153,154,155]. China has simplified the approval process for crops without foreign genes, while maintaining environmental and health risk assessments [156,157]. This creates challenges for international trade and harmonization of regulations. Ethical aspects include possible unintended effects of editing, impact on biodiversity, and concentration of biotechnological resources in the hands of large companies. For consumers, transparency in labeling, awareness, and trust in the safety of the technology remain key. Increased openness, scientific justification of risks, and active dialog with society are necessary for the successful integration of CRISPR-edited vegetables into agriculture.

## Data Availability

No new data were created or analyzed in this study. Data sharing is not applicable to this article.

## Abbreviations

The following abbreviations are used in this manuscript:

CRISPR	Clustered Regularly Interspaced Short Palindromic Repeats
Cas	CRISPR-associated protein
DSBs	Double-stranded breaks
NHEJ	Non-homologous end joining
HDR	Homology-directed repair
FHB	Fusarium head blight
BSMV	Barley mosaic virus
VIGS	Virus-induced gene silencing
RNP	Ribonucleoprotein
SNB	Septoria nodorum blotch
*MLO*	Powdery mildew locus O
CRISPRi	CRISPR interference
BYDV	Barley yellow dwarf virus
FAO	Food and Agriculture Organization of the United Nations
*DMR6*	Downy mildew resistance 6
*DND1*	Defense no death 1
*DCL1*	Dicer-like1
*NBS-LRR*	Nucleotide-binding site
LRR	Leucine-rich repeat
*XSP10*	Xylem sap protein 10
*SlSAMT*	Salicylic acid methyltransferase
LTP	Lipid transfer protein
SA	Salicylic acid
MeSA	Methyl salicylate
SAM	S-adenosyl-L-methionine
*SlPG2a*	Polygalacturonase
*SlPL*	Pectate lyase
GMOs	Genetically modified organisms
